# Understanding the Patient Experience with Twice-Nightly Sodium Oxybate Therapy for Narcolepsy: A Social Listening Experiment

**DOI:** 10.3390/brainsci14121189

**Published:** 2024-11-26

**Authors:** Maria Picone, Frederik Ascencion, Matthew Horsnell, Enming Zhang, Lauren Dougherty, Christopher DeFelice, Maurice Flurie, Rachelle Cook, Anne Marie Morse, Luis E. Ortiz, E. Robert Wassman, Jennifer Gudeman

**Affiliations:** 1 TREND Community, Philadelphia, PA 19102, USA; 2 PWN4PWN, Tampa, FL 33602, USA; 3 Geisinger Commonwealth School of Medicine, Geisinger Medical Center, Janet Weis Children’s Hospital, Danville, PA 17822, USA; 4 Johns Hopkins Medical Institutions, Johns Hopkins All Children’s Hospital, St. Petersburg, FL 33701, USA; 5 Avadel Pharmaceuticals, Chesterfield, MO 63005, USA

**Keywords:** social listening, narcolepsy, sodium oxybate, patient voice, natural language processing

## Abstract

Background/Objectives: Narcolepsy is a chronic neurologic disorder associated with substantial challenges that affect the social, emotional, and financial quality-of-life domains. A social listening analysis and structured survey were conducted to better understand the candid perspective of people with narcolepsy (PWN) and their experience with twice-nightly sodium oxybate (SXB). Methods: To characterize conversations and experiences in narcolepsy communities where SXB was mentioned, a social media analysis was conducted from August 2011 to October 2022. A structured survey was administered to PWN taking oxybate therapy from October 2022 to November 2022. Results: From the social media analysis, the largest topic was related to “cataplexy”, with 537 posts/comments. The most frequent term was “xyrem”, with 22,200 mentions. Of the 87 survey respondents, 85 respondents had narcolepsy, 75.3% (64/85) reported missing their second dose of immediate-release SXB or mixed-salt oxybates, and 58.8% (50/85) of respondents reported that they took their second dose of oxybate > 4 h after the first dose. Respondents reported poor sleep quality as the greatest effect or issue experienced after missing their second oxybate dose. When respondents were asked whether they had ever been injured after waking to take their second oxybate dose, 31.8% (27/85) of respondents reported an injury. Conclusions: PWN taking twice-nightly oxybates often report inconsistent adherence to the prescribed dosing, which results in negative consequences in their lives. This research provided an anonymized forum for PWN to voice challenges with middle-of-the-night awakenings that they may be reluctant to explain to their clinician.

## 1. Introduction

Narcolepsy is a chronic neurologic disorder in which the brain cannot regulate sleep–wake cycles; symptoms affect all 24 h of the day [1]. Narcolepsy affects approximately 1 in 2000 people [2]. The defining feature of narcolepsy is excessive daytime sleepiness (EDS) [3], which is frequently the most disabling feature for people with narcolepsy (PWN) [1]. However, symptoms of narcolepsy extend into nighttime sleep and include sleep paralysis, sleep-related hallucinations, and disrupted nighttime sleep (DNS). Sleep paralysis is the persistence of rapid eye movement (REM) sleep atonia [4], resulting in the inability of PWN to move or speak during sleep–wake transitions [1]. Sleep-related hallucinations, known as hypnagogic or hypnopompic hallucinations, are experiences in which PWN falsely hear, see, or feel something while falling asleep or upon awakening [1]. REM sleep-related hallucinations can be associated with sleep paralysis [5]. DNS is best characterized by the innate sleep state instability found in narcolepsy but has multifactorial contributors to the lack of continuous restorative sleep [6,7]. The last of the 5 symptoms, cataplexy, is specific for narcolepsy type 1 (NT1); it is defined as a sudden loss of voluntary muscle control in response to an emotion [1]. However, this definition fails to include consideration of the active motor phenomena that can occur [8], as well as PWN who experience similar episodes of sudden muscle tone loss devoid of a recognized emotion [9]. Generalized cataplexy causes PWN to collapse fully while remaining conscious; partial cataplexy may present more subtly as facial twitching or drooping, dropping items, or stumbling [6]. While the etiology of NT1 is recognized as a loss of the neurotransmitter orexin, the etiology of narcolepsy without cataplexy, also referred to as narcolepsy type 2, is less clear [1].

In September 2013, the US Food and Drug Administration (FDA) convened a Patient-Focused Drug Development (PFDD) meeting to gather perspectives from PWN to better understand the effect of the disease on their daily life and to help inform future drug development programs [10]. As the FDA report noted, for PWN who experience episodes of cataplexy, hallucinations, or sleep paralysis, “the uncontrollable and often unpredictable loss of control can be terrifying”. Substantial challenges were ascribed to narcolepsy, as PWN described negative effects impacting the social, emotional, and financial domains.

At the time of the 2013 PFDD, the only FDA-approved treatments for narcolepsy included stimulants and Xyrem^®^, the brand name of sodium oxybate (SXB) [10]. While some PWN commented on the transformational effect of SXB, others mentioned the challenges associated with the dosing regimen, which requires PWN to take 1 dose at bedtime and wake up to administer a second dose 2.5–4 h later [11]. PWN expressed their desire for more effective treatment options, as they described not wanting narcolepsy to “control” them but instead to control their narcolepsy symptoms.

At the same time as the FDA PFDD, a pharmaceutical company was starting a drug development program for an extended-release SXB, formulated to be taken once at bedtime (ON-SXB). This formulation of SXB was designed to provide comparable exposure of medication during the nocturnal sleep period to that provided by 2 immediate-release (IR) doses [12]. This medication, ultimately approved in May 2023 as LUMRYZ™ [13,14], had a previously anticipated approval date of October 2021 [15]. However, a patent dispute delayed the approval [16], preventing PWN from accessing this once-nightly oxybate formulation [17].

During this delay, the pharmaceutical sponsor sought to validate their assumptions by engaging directly with PWN to better understand their unmet needs. PWN anecdotes confirmed substantial challenges with waking up in the middle of the night for an extended period of time. These anecdotes were supported by data generated from an open-label study in which participants were able to switch from the twice-nightly to the once-nightly oxybate regimen [18]. Despite the challenges noted by PWN, some sleep clinicians may normalize middle-of-the-night dosing of oxybates, as this was the only option for >20 years [11,14]. Upon further probing to understand the discordance between PWN and clinicians, PWN anecdotes revealed that patients were reluctant to voice challenges with waking up in the middle of the night to take their second dose of medication. To better understand the candid PWN perspective, a social media listening and structured survey study was undertaken.

## 2. Materials and Methods

### 2.1. Overview

The goal of this study was to characterize conversations and experiences in narcolepsy communities when SXB is mentioned. This study was reviewed and approved by an Institutional Review Board. First, a social media analysis was conducted. Open-source natural language processing analysis techniques were implemented to identify topics and their prevalence discussed in social media posts and comments across multiple online narcolepsy communities. A structured survey was then administered to better understand patient experiences with taking oxybate therapy for narcolepsy. The social media analysis and survey were conducted by TREND Community, a community-powered data analytics company. TREND Community uses machine learning and natural language processing techniques to analyze online conversations of partnered communities of people with rare, chronic, and emerging diseases, as well as their caregivers, to report insights on the impact and unmet needs of the disease.

### 2.2. Data Sources

#### 2.2.1. Social Media

Data included in the analysis were 2 social media sources: a private Facebook group, People with Narcolepsy 4 People with Narcolepsy (PWN4PWN), and a public subreddit, r/narcolepsy. PWN4PWN included 25,596 posts/comments and 1312 members. The r/narcolepsy source included 229,564 posts/comments and 15,358 members. Posts/comments spanned from August 2011 to October 2022. Conversations discussing SXB were isolated for analysis, resulting in a sample of 26,455 posts/comments from August 2011 to October 2022. Pattern matching was used to identify conversations discussing SXB. A subset of terminology associated with SXB (e.g., “oxybate”, “xyrem”, “xywav”, “ghb”, and “gamma hydroxybutyrate”) was used to isolate posts/comments for topic analysis. Topics of interest were identified for further exploration in a survey.

#### 2.2.2. Survey

Survey respondents were recruited by the TREND Community. A survey using Google Forms was fielded to PWN4PWN, a private Facebook group comprising 1312 people living with narcolepsy, from 12 October 2022 to 6 November 2022. Inclusion criteria required respondents to be age ≥ 18 years, live in the United States, be a patient or caregiver of a person with a narcolepsy diagnosis, and currently be taking or have previously taken oxybate therapy, such as Xyrem^®^ or Xywav^®^. Overall, 89 people began the survey, but 1 caregiver dropped out on their own and 1 respondent was terminated because they had idiopathic hypersomnia, resulting in 87 respondents. Respondents who completed the survey received a $10 Amazon gift card.

### 2.3. Data Processing

Topic analysis was conducted to reveal latent topics and themes discussed in SXB conversations. BERTopic was used to identify topics in conversations [19]. BERTopic is a topic modeling approach that produces document (i.e., post or comment) embeddings with pretrained transformer language models. Clustering techniques are then implemented to derive related documents and class-based term frequency-inverse document frequency procedures, which are used to characterize the most associated terms in each identified topic. The model in the current study was trained with the “all-mpnet-base-v2” sentence transformer model to derive embeddings, Uniform Manifold Approximation and Projection for dimensionality reduction [20], and hierarchical, density-based clustering of related language to isolate individual topics [21]. Topics with other medications (e.g., Adderall^®^) included as associated terms were not considered for analysis.

## 3. Results

### 3.1. Social Listening

**Figure 1:**
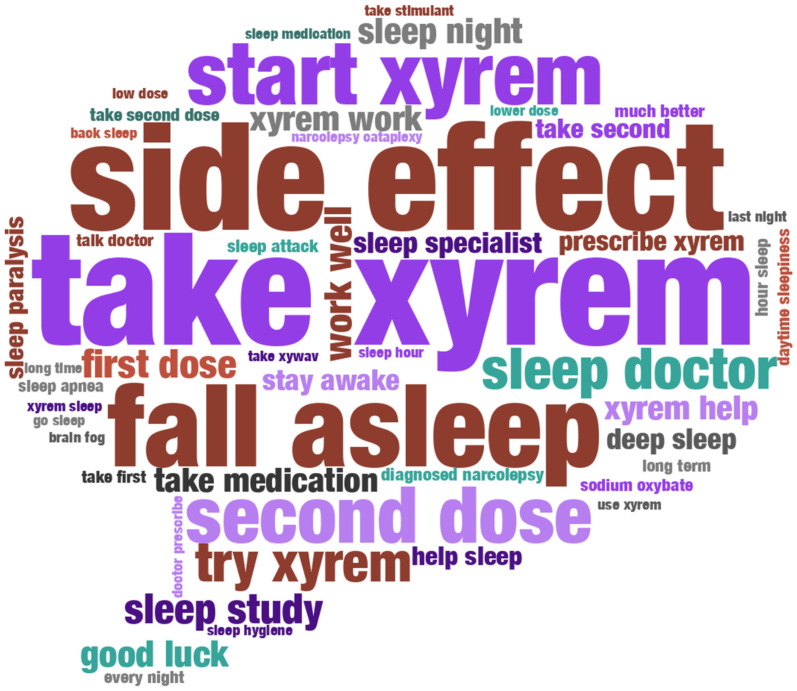
Top 50 Bigrams and Trigrams “Sodium Oxybate” Posts/Comments. Word cloud of the top 50 most frequent terms used in conversations discussing sodium oxybate.

Among the top 200 terms in SXB conversations, after removing common stop words (e.g., “the”, “it”, “for”, and “at”), the mean (SD) term frequency was 2429.47 (1908.36). The most frequent term was “xyrem”, with 22,200 mentions. While “xywav” appeared in the top 20 terms with 4456 mentions, the least frequent term was “kind”, with 1112 mentions. A word cloud (Figure 1) was generated using the 50 most frequent bigrams and trigrams from SXB conversations, after filtering out generic temporal phrases (e.g., “year ago”, “month ago”), common expressions without context (e.g., “make sure”, “want try”), and redundant combinations (e.g., “xyrem xywav”). The remaining phrases represent specific aspects of medication safety (“side effect”), dosing management (“first dose”, “second dose”, “low dose”), medical experiences (“sleep doctor”, “sleep study”), symptoms (“sleep paralysis”, “daytime sleepiness”), and treatment outcomes (“work well”, “help sleep”). The size of each phrase corresponds to its frequency, ranging from “take xyrem” (3095 mentions) to “xyrem sleep” (350 mentions).

**Figure 2:**
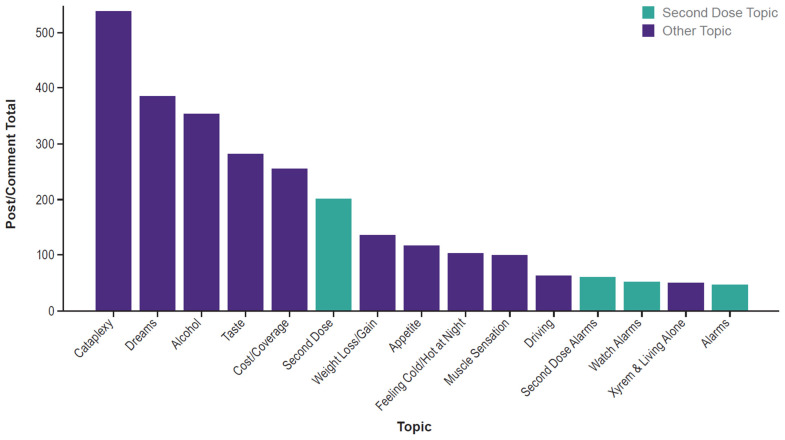
Most Prevalent Topics Discussed Across Social Media Posts and Comments. Bar chart illustrating the most frequent topics mentioned across social media posts and comments.

Topic analysis revealed 15 definable topics with ≥ 45 posts/comments (Figure 2). The largest topic was related to “Cataplexy”, with 537 posts/comments, and the smallest was “Alarms”, with 46 posts/comments. Four of the top 15 topics were identified as potentially relating to the second (nightly) dose of SXB medications: “Second Dose” (191 posts/comments), “Second Dose Alarms” (61 posts/comments), “Watch Alarms” (51 posts/comments), and “Alarms” (46 posts/comments). Individuals described the difficulties they have experienced with the second dose, as a summarized quote from a user said the following quotation from the “Second Dose” topic: “...[I] wake up as soon as the second dose wears off and cannot get back to sleep after”. Another user, identified in the “Second Dose Alarms” topic, described SXB as a “miracle drug” that “transformed” their life despite the challenge of waking up for a second dose.

### 3.2. Survey

Of the 87 survey respondents, 85 (97.7%) were diagnosed with narcolepsy and 2 (2.3%) were caregivers (Table 1). Most respondents with narcolepsy were female (75.3%), had NT1 (51.8%), and were currently taking mixed-salt oxybates (52.9%).

**Figure 3:**
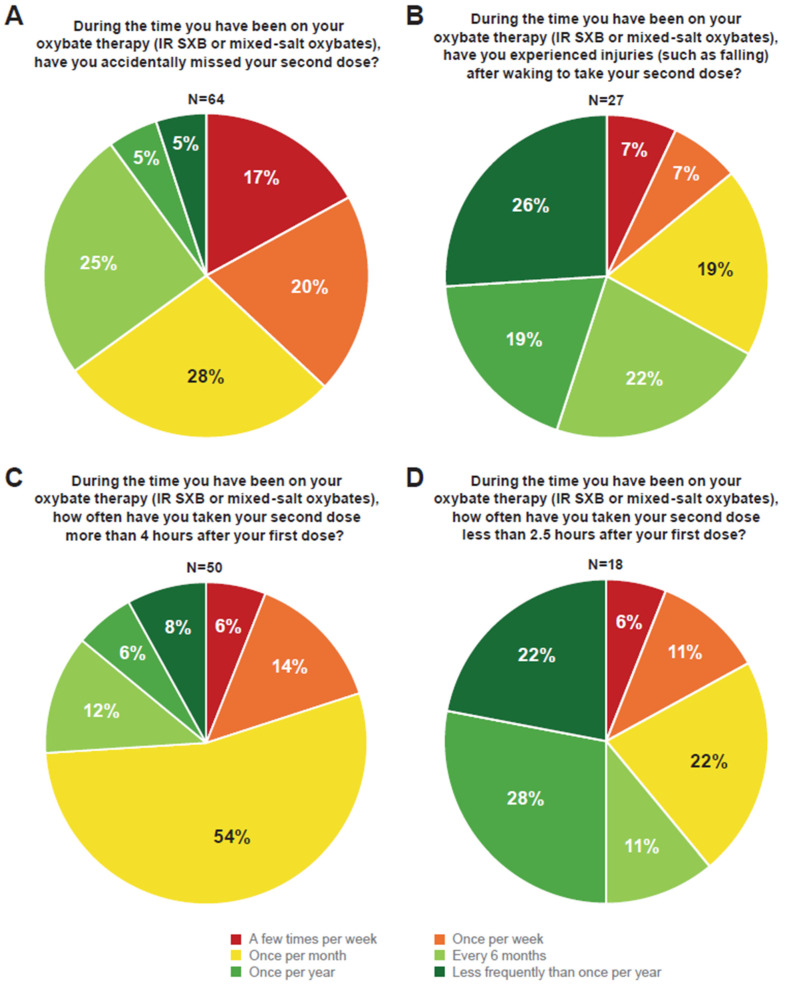
Survey Questions and Responses. IR SXB, immediate-release sodium oxybate. Pie charts of survey questions and responses. (**A**) Frequency of missed second doses of IR SXB or mixed-salt oxybates. (**B**) Frequency of injuries after waking to take a second dose of IR SXB or mixed-salt oxybates. (**C**) Frequency of taking a second dose of IR SXB or mixed-salt oxybates > 4 h after the first dose. (**D**) Frequency of taking a second dose of IR SXB or mixed-salt oxybates < 2.5 h after the first dose.

The majority (64/85; 75.3%) of respondents with narcolepsy reported missing their second dose of IR SXB or mixed-salt oxybates during the time they were on oxybate therapy. Of these 64 respondents, 23 (35.9%) reported missing their second dose more often than once per month (Figure 3A). As a result of missing their second dose, respondents most frequently reported poor sleep quality, excessive or increased daytime sleepiness, and decreased next-day productivity, affecting their next-day functioning (Table 2). Seven respondents specifically said they have trouble waking in the morning after missing their second dose; 10 other respondents made comments that implied trouble waking up (e.g., tardiness, absences from work/school, and missing alarms). One individual described difficulties in taking their second dose on a regular schedule, stating, “I was unable to take the second dose of IR SXB the same time every night, and sometimes not at all. While staying awake during the day is difficult, waking up is the hardest thing I do all day”.

When asked whether they had ever been injured after waking to take their second oxybate dose, 27/85 (31.8%) respondents reported an injury. Five (18.5%) of these 27 respondents reported that an injury occurred once per month, and 4/27 (14.8%) reported that an injury occurred once per week or more often (Figure 3B). Respondents reported falls, bumps, bruises, and black eyes as a result of waking to take their second dose. One respondent reported that they “knocked over [the] CPAP onto [the] floor and walked around confused, bumping into the wall and/or stubbing toes looking for [the] second dose and forgetting it was next to the bed all along”. Another individual reported, “on multiple occasions, I have fallen asleep while on the toilet in between doses. I already touched on how brain fog can hinder and be dangerous when mixing and preparing nighttime doses of medication, but I have also experienced injuries and extreme confusion. A few examples are stumbling through doorways and hallways while trying to get up to take my second dose”. More serious injuries, such as stitches, pulled muscles, and a need for physical therapy owing to a neck and shoulder injury, were also reported. One respondent described an incident in which they took their second dose too soon and ended up in the emergency room. Concussions have been reported; as one respondent stated, “[I received] frequent minor injuries (mild bumps and bruises). I have had several concussions that caused more serious issues and required treatment from a concussion clinic due to lingering concussion symptoms and periodic reinjury due to additional concussions under the same circumstances. Overall, the concussions have taken a big toll on my overall functioning and mental health”. When asked to provide details about other issues experienced, some respondents mentioned bed partners. One person spoke about the fear of disrupting a partner, “Worried about disrupting bed partner with alarm I have to set to take second dose on time”. Another spoke of a concerned partner, “My partner is troubled by my behavior sometimes when I wake for my second dose”.

More than half (50/85; 58.8%) of respondents reported that they had taken their second dose of oxybate > 4 h after the first dose. Of these 50 respondents, 27 (54.0%) reported taking their dose late once per month (Figure 3C). The most common effects or issues due to taking their second dose late included missing activities, grogginess, or difficulty functioning the next day. Tardiness to work or school resulting in disciplinary action was a common theme, as respondents reported, “extended brain fog caused frequent late arrivals at work resulting in termination” and “missed morning activities and impacting of my daily life things for sure... it has gotten me in serious trouble from time to time where I’ve gotten written up because of my tardiness”. Late doses have also contributed to safety concerns. One respondent stated, “Sometimes, the brain fog during oxybate therapy lasted longer than anticipated, and a few times, this resulted in my driving impaired and damaging my vehicle”. One individual described the unique challenge of timing and measuring their IR SXB doses while already sleepy, stating, “Making sure I get the second dose has been a huge hurdle for me. When I don’t get in both doses, it disrupts my schedule. This leads to waking up late or in a cloud of brain fog. This creates a cycle of missed doses, causing more missed doses. Trying to measure out both doses at night and figure out timing is made more difficult because of the sleepiness”.

Eighteen of 85 (21.2%) respondents reported taking their second dose < 2.5 h after the first dose compared with 50/85 (58.8%) respondents who took their second dose > 4 h after the first dose. Of the 18 who took their second dose early, 4 (22.2%) respondents reported this happening once per month, 2 (11.1%) reported this happening once per week, and 1 (5.6%) reported this happening a few times per week (Figure 3D). When asked to describe any effects or issues experienced after taking the second dose early, some respondents expressed fear and anxiety, while others shared that they felt confused or disoriented. Some respondents shared feelings of physical discomfort after taking their second dose early, as 1 respondent shared that they experienced “severe digestive upset, headache, increased EDS, and worsening of symptoms”. A more serious reaction was reported by 1 respondent who wrote, “I was terrified once I looked at the clock and realized what I had done, ended up in the ER, courtesy of my mom, knocked out and with a respiration rate of 5 breaths per minute”.

Respondents were asked whether they agreed that a once-nightly dose of SXB in a premeasured dosing packet would be safer than the currently available oxybate formulations; 66/87 (75.9%) strongly agreed or agreed. Prior to the FDA approval of ON-SXB, one respondent stated, “I am awaiting approval of once-nightly sodium oxybate because no other medicinal or lifestyle regimens have allowed me to complete my ADLs [activities of daily living] without significant struggle. I am so sad that I cannot spend the time I would like to with my 4-year-old son and husband due to my EDS, and [I] am not able to take IR SXB anymore due to missing the second dose too frequently due to shutting off my alarms, and going back to sleep, or hallucinating and thinking I took my second dose when I really did not”. Another respondent described an administration error with IR SXB, as they reported, “Accidentally poured a liquid that wasn’t plain water into the cup. Accidentally poured water into another different medication bottle, ruining an entire [month’s] script”. The convenience of a premeasured packet for travel was noted by 1 individual who stated, “Another advantage of prepackaged packets is not having to carry liquids around during travel. During a trip to Vietnam, I had the bottle top become slightly loose and ended up having some of the liquid medication leak out into my luggage”.

## 4. Discussion

Prior research in other disease states has shown adherence to be inversely related to dosing frequency [22,23,24]. Studies have also demonstrated that high adherence to recommended dosing regimens leads to the optimization of symptom management and disease control [25,26]. In addition to these findings, the present analysis describes potential harms experienced when there is inconsistent adherence with dosing regimens. 

These data contribute to clinical practice by underscoring the clinical misperception that may occur regarding the ease and feasibility of consistent dosing. Findings from this study demonstrate that adhering to the prescribed dosing of twice-nightly oxybates is challenging, as evidenced by issues with injuries related to the second dose, missed doses, and improper timing between doses (i.e., too early or too late). Survey participants reported experiencing negative consequences in their lives, such as increased daytime sleepiness, reduced productivity, and injuries. While less frequent, 18 of the 85 respondents reported taking their second dose < 2.5 h after the first dose; this phenomenon has been described elsewhere as leading to serious complications, including hospitalization [27,28,29]. Findings for term frequency illustrate the primary medications discussed were Xyrem and Xywav. Naturally occurring topics in SXB conversations were often directly or indirectly related to the second nightly dose, indicating its relevance to the community and warranting further exploration via a structured survey. While the impact on bed partners was not actively solicited, given the relevance of the comments obtained passively, future research specifically into bed partner impact for narcolepsy in general and middle-of-the-night dosing with twice-nightly oxybates should be pursued.

Patients have a myriad of reasons for not being fully candid when discussing chronic medications with their clinicians, including a desire to “please” their doctor, guilt if they are not able to follow orders, fear of being labeled as “noncompliant” and the potential negative impacts on future care as a result, a feeling of the time constraint reality of typical healthcare provider interactions, and a feeling that their current treatment is “good enough”. Clinicians may consider asking their patients whether they are having any difficulty with the second dose to understand whether their experiences are similar to, or different from, those described in these data. In addition, clinicians may need to create a “safe space” for PWN to understand their experience better rather than relying on patients to volunteer information.

SXB is the sodium salt of γ-hydroxybutyrate (GHB). GHB is an important endogenous precursor and metabolite of γ-aminobutyric acid [30], which has been implicated in the regulation of sleep [31,32]. However, due to the unfortunate history of illicit GHB use [33], clinicians and PWN may have concerns about the potential risks. Specifically, some PWN may worry about losing access to SXB if it is not taken as prescribed exactly. All FDA-approved oxybate medications require a Risk Evaluation and Mitigation Strategy (REMS) program [34,35]. These programs include “elements to assure safe use” [36]; specifically, the distribution of oxybates is limited to REMS-certified specialty pharmacies that are required to complete Patient Counseling Checklists [34,35]. Only clinicians who are REMS certified are permitted to prescribe oxybates; PWN must also be enrolled in the REMS program to ensure both stakeholder groups understand the additional risks associated with oxybates.

REMS programs are FDA-mandated safety programs; they are required when a potential risk with medication may preclude FDA approval, unless medication-specific risk mitigation plans are operationalized, including ongoing reports to the FDA to demonstrate the effectiveness of the REMS program [37]. For oxybates, the risks include the potential for misuse, abuse, or diversion and central nervous system depression, which may be exacerbated by alcohol and sedative hypnotics (both contraindicated with oxybates) [34,35].

REMS programs for oxybates are necessary and help to keep PWN safe. An unintended consequence of oxybate REMS programs may be that PWN are reluctant to relay information that they believe could jeopardize their access to a medication that they rely upon, such as inconsistent usage or injuries obtained while taking the medication, as observed in this study. Thus, creating a safe space in which anonymized responses could be provided is critical to understanding PWN perspectives.

Our study explores the ability of social media listening as an additional, valuable tool for gaining insights into the patient journey, including issues related to adherence with twice-nightly dosing of IR SXB. Social media support groups have become prominent platforms where individuals discuss their conditions, offering real-time data that complement traditional research methods. Social media listening allows researchers to capture organic discussions, providing immediate insights into patient experiences and concerns. This approach accelerates the identification of key issues, such as challenges with the second nightly dose, enabling timely interventions and focused research efforts. By incorporating social media listening into our research methodology, we aim to expedite the generation of insights, potentially enhancing the efficiency and effectiveness of our research efforts. Future research could use signals identified via social listening to explore topics that are important to PWN.

While social media listening may facilitate quicker development of targeted solutions, which aim to improve patient outcomes and satisfaction, it does have limitations. Participants were self-selected to those with access to technology and who could speak English, which potentially skews the sample and may not be representative of the broader narcolepsy population; certain groups may be underrepresented. Additionally, this study draws the assumption that participation in these social media spaces reflects that the participating individual is affected by narcolepsy; however, shared information on social media is unverifiable, which affects data reliability. PWN who had difficulty with the middle-of-night dose were more likely to participate in the survey, which may limit the generalizability of the findings to the overall population.

## 5. Conclusions

In conclusion, findings from this study validate anecdotes from PWN that chronically waking up in the middle of the night is associated with many negative consequences. PWN may not be fully candid when discussing concerns with their clinician for many reasons. Clinicians should create a safe space for PWN to understand their experience better rather than relying on patients to volunteer information to understand whether their experiences are similar to or different from the experiences described.

## Figures and Tables

**Table 1:** Survey Respondent Characteristics.

Characteristic, n (%)	Number of Respondents (N = 87)
Caregiver	2 (2.3)
Patient diagnosed with narcolepsy	85 (97.7)
Sex	
Female	64 (75.3)
Male	19 (22.4)
Intersex/prefer not to answer	2 (2.4)
Narcolepsy type	
NT1	44 (51.8)
NT2	39 (45.9)
Unsure	2 (2.4)
Prior or current use of IR oxybate	
Currently taking IR SXB	19 (22.4)
Currently taking mixed-salt oxybates	45 (52.9)
Previously taken IR SXB	34 (40.0)
Previously taken mixed-salt oxybates	11 (12.9)

IR, immediate-release; NT1, narcolepsy type 1; NT2, narcolepsy type 2; SXB, sodium oxybate. Table describing demographic and disease characteristics of survey respondents.

**Table 2:** Effect of Missing Second SXB Dose.

Effect of Missing Second SXB Dose, n	Number of Mentions
Poor sleep quality	26
Increased daytime sleepiness	20
Excessive daytime sleepiness	10
Decreased next-day productivity	9
Worsening symptoms	8
Brain fog	7
Missed time at work or school	7
Increased irritability	5
Lack of energy	4
Headache and/or migraine	3
Increased cataplexy	2
Increased fatigue	2

SXB, sodium oxybate. Table of survey responses describing the effect of missing the second dose of SXB.

## Data Availability

The datasets generated and/or analyzed during the current study are not publicly available due to data privacy and legal considerations.
